# Core Competencies in Clinical Neuropsychology as a Training Model in Europe

**DOI:** 10.3389/fpsyg.2022.849151

**Published:** 2022-03-31

**Authors:** Mary H. Kosmidis, Sandra Lettner, Laura Hokkanen, Fernando Barbosa, Bengt A. Persson, Gus Baker, Erich Kasten, Amélie Ponchel, Sara Mondini, Nataliya Varako, Tomas Nikolai, María K. Jónsdóttir, Aiste Pranckeviciene, Erik Hessen, Marios Constantinou

**Affiliations:** ^1^Lab of Cognitive Neuroscience, School of Psychology, Aristotle University of Thessaloniki, Thessaloniki, Greece; ^2^Association for Neuropsychology Austria, Bad Häring, Austria; ^3^Department of Psychology and Logopedics, Faculty of Medicine, University of Helsinki, Helsinki, Finland; ^4^Laboratory of Neuropsychophysiology, Faculty of Psychology and Education Sciences, University of Porto, Porto, Portugal; ^5^Department of Psychology, Linnaeus University, Växjö, Sweden; ^6^Clinical Neuropsychology - Molecular and Clinical Pharmacology, University of Liverpool, Liverpool, United Kingdom; ^7^Department of Psychology, MSH University of Applied Sciences and Medical University, Hamburg, Germany; ^8^French Federation of Psychologists and Psychology (FFPP), French Organization of Psychologists Specialized in Neuropsychology OFPN, GHU Paris Psychiatrie & Neurosciences, Paris, France; ^9^Department of Philosophy, Sociology, Education and Applied Psychology, University of Padua, Padua, Italy; ^10^Psychological Methodology Department, Faculty of Psychology, Lomonosov Moscow State University, Moscow, Russia; ^11^Research Center of Neurology, Moscow, Russia; ^12^Psychological Institute of the Russian Academy of Education, Moscow, Russia; ^13^Department of Neurology, Charles University in Prague, Prague, Czechia; ^14^Department of Psychology, School of Social Sciences, Reykjavik University, Reykjavík, Iceland; ^15^Department of Health Psychology, Lithuanian University of Health Sciences, Kaunas, Lithuania; ^16^Department of Psychology, University of Oslo, Oslo, Norway; ^17^Department of Social Sciences, University of Nicosia, Nicosia, Cyprus

**Keywords:** expertise, psychology specialty, entry-level professional competence, clinical neuropsychology, training

## Abstract

The multitude of training models and curricula for the specialty of clinical neuropsychology around the world has led to organized activities to develop a framework of core competencies to ensure sufficient expertise among entry-level professionals in the field. The Standing Committee on Clinical Neuropsychology of the European Federation of Psychologists’ Associations is currently working toward developing a specialty certification in clinical neuropsychology to establish a cross-national standard against which to measure levels of equivalency and uniformity in competence and service provision among professionals in the field. Through structured interviews with experts from 28 European countries, we explored potential areas of core competency. Specifically, questions pertained to the perceived importance of a series of foundational, functional, and other competencies, as well as current training standards and practices, and optimal standards. Our findings revealed considerable agreement (about three quarters and above) on academic and clinical training, despite varied actual training requirements currently, with fewer respondents relegating importance to training in teaching, supervision, and research (a little over half), and even fewer to skills related to management, administration, and advocacy (fewer than half). European expert clinical neuropsychologists were in agreement with previous studies (including those conducted in the United States, Australia, and other countries) regarding the importance of sound theoretical and clinical training but management, administrative, and advocacy skills were not central to their perspective of a competent specialist in clinical neuropsychology. Establishing a specialty certificate in clinical neuropsychology based on core competencies may enable mobility of clinical neuropsychologists across Europe, and, perhaps, provide an impetus for countries with limited criteria to reconsider their training requirements and harmonize their standards with others.

## Introduction

Recent decades have witnessed a rapid increase in the number of training programs and professionals in clinical neuropsychology across the globe. Concurrently, research in the field is increasingly broadening our knowledge base regarding brain-behavior relationships and underlying mechanisms in varied disorders affecting cognition, emotion, and behavior. While some countries have developed well-defined criteria and rigorous training requirements (e.g., the United States, the United Kingdom, Australia, Norway, Netherlands, and Austria) to establish the competency of new professionals in this specialty field, many others either have not, or have done so to a lesser degree. Given the ever-evolving nature of clinical neuropsychology as a professional specialty and scientific area, as well as technological and other advances relevant to the practice of neuropsychology, establishing a list of areas of competency considered as core elements for new specialists entering the profession would appear paramount.

Indeed, even the United States, which originally established criteria for training in clinical neuropsychology over two decades ago (The Houston Conference on Specialty Education and Training in Clinical Neuropsychology policy statement, Hannay et al., 1998), has recently re-evaluated, expanded and updated these criteria. Specifically, the Clinical Neuropsychology Synarchy (CNS), the convenor of United States organizations relevant to clinical neuropsychology, established a competency-based taxonomy, congruent with recent initiatives of the American Psychological Association (APA) to standardize education and training in the specialty areas of professional psychology (Kaslow, 2004). This resulted in three documents describing essential criteria of training in clinical neuropsychology (Smith, 2019): “Taxonomy for Education and Training in Clinical Neuropsychology” (Sperling et al., 2017), “Entry Level Competencies for Clinical Neuropsychology” and a document regarding competencies to be gained at the post-doctoral residency level (Smith, 2019).

Similarly, efforts within Europe to streamline education and professional training of psychologists in general has resulted in certification based on having met a standard level of academic and professional training. This certification, known as the European Certificate in Psychology (EuroPsy) provides assurance to potential clients or employers beyond national borders, of the required competency level of the psychologist, based on equivalence of standards across Europe. This is in accordance with the stated mission and aims of the European Federation of Psychologists’ Associations (EFPA), which, among others, seeks “to promote the development, dissemination, and application of psychology in all of its forms and to further develop psychology (academically, scientifically, and professionally), including the improvement of the quality of the profession and its legal status in each member state” (European Federation of Psychologists’ Associations, 2015b). To overcome, and in fact, accommodate cross-national differences in training paradigms, while concomitantly ensuring equivalent and uniform qualifications and levels of expertise when entering the profession, activities geared toward developing specialty certification are based on a ‘competence’ approach. This entails a common framework and view of the profession and some specialty areas of psychology.

In some European countries, training standards and competency requirements for entry into the professional specialty area of clinical neuropsychology have been clearly defined for quite some time. We present brief descriptions of some of these countries’ requirements below.

### Norway

The specialty in clinical neuropsychology was established in Norway in 1987. A candidate can enter specialist training in clinical neuropsychology only after completing the Norwegian 6-year university-based general psychology course of study leading to certification as a psychologist. The duration of specialist training is a minimum of 5 years and requires at least four full work-years in supervised clinical neuropsychology training with both children and adults, for a minimum of 1 year of practice with each age category. During specialist education, the candidate must meet a representative variety of patients with brain damage or disorder, both with regard to diagnostic categories and severity of condition. At least two of the 4 years of full-time neuropsychological work need to take place in departments of neurology, neurosurgery, physical medicine, pediatrics, rehabilitation, epilepsy, or similar. The other 2 years of full-time neuropsychological practice can be in child psychiatry, adult psychiatry, geriatrics, etc. It is required that all practice includes close and continued collaboration with a team of relevant medical specialists. During the clinical training, the specialization requires 3 years of courses (1 year in general clinical psychology and 2 years in clinical neuropsychology) and completion of a scientific paper^1^.

The aim of the Norwegian specialization is that candidates shall integrate the core competencies outlined in Hessen et al. (2018) that are characteristic of some of the most developed training programs in clinical neuropsychology throughout the world. That is (1) to gain in-depth knowledge of general psychology, including clinical psychology, and ethical and legal standards; (2) to develop expert knowledge of clinically relevant brain-behavioral relationships; (3) to acquire comprehensive knowledge and skills in related clinical disciplines; (4) to achieve in-depth knowledge and skills relevant to neuropsychological assessment, including decision-making and diagnostic competency according to current classification of diseases; (5) to acquire competencies in the area of diversity and culture in relation to clinical neuropsychology; (6) to develop competency in communicating neuropsychological findings and test results to relevant and diverse audiences, and, finally, (7) to gain solid knowledge and skills relevant to psychological and neuropsychological intervention, including treatment and rehabilitation.

### United Kingdom

In the United Kingdom, qualification for the specialty area requires 2 years of supervised work in the field of clinical neuropsychology, following a doctoral degree in clinical or educational psychology. The Specialist Register of Clinical Neuropsychologists [SRCN] is the gold standard for registration for clinicians practicing in neuropsychology and provides a quality mark for their expertise, as well as assurance that the skills and experience of an individual have been validated and are of the appropriate standard. Candidates for entry to the SRCN core have to demonstrate that they possess the necessary clinical and academic experience to meet the requirements for entry into the Register.

To join the register, clinicians must undergo additional specialist post-doctoral training in brain injury and neurological illness and gain substantial experience in applied neuropsychological assessment and treatment. This involves a minimum period of clinical supervision, academic study, and submission of a clinical portfolio. There are a number of prescribed routes for the academic component, including self-study and formal university courses. Consideration is currently being given to a fast-track option via a doctorate in clinical neuropsychology (The British Psychological Society, 2019).

Additionally, the British Psychological Societies Division of Neuropsychology [DoN] has devoted much effort to describing core competency frameworks for adult neuropsychology and child neuropsychology. The DoN has published the ‘‘Competency Framework of the United Kingdom Clinical Neuropsychology Profession 21.05.2012.’’ The purpose of developing a competency framework was as follows: to develop standards for Clinical Neuropsychology practice in the United Kingdom; to objectively regulate entry to the specialty area of Clinical Neuropsychology in the United Kingdom (entry to the Division of Neuropsychology’s specialist register); to identify the expected competencies developed by candidates undertaking the Qualification in Clinical Neuropsychology (QiCN). The four domains of the framework include underpinning knowledge, clinical work, communication, and personal and professional practice^2^.

### Netherlands

Requirements in the Netherlands include 3-year post graduate training involving at least 4,860 h (National Education Renewal Working Group, 2018) of practical training (about 3,240 h for supervised clinical work and 1,360 h teaching), with about 260 h of supervision, and at least 600 h of courses, 760 h of reading the literature and practical assignments. The Dutch competence-based training applies the model of the Canadian Medical Education Directives for Specialists (CanMEDS^3^), in which the key roles are defined as Medical Expert, Communicator, Collaborator, Manager, Health Advocate, Scholar, and Professional. These roles have been adapted for psychologists, and detailed competencies and indicators for each are presented in the Educational plan and Workbook for clinical neuropsychology (De Federatie van Gezondheidszorgpsychologen en Psychotherapeuten, 2019^4^). They include the following areas of competency: providing optimal diagnosis and treatment procedures based on the relevant scientific literature; development of effective relationships with patients, including communicating with them regarding decisions related to their treatment; consultation with other relevant professionals; comprehension and translation of research into applied diagnostic and treatment methods; conduction or promotion of the dissemination of scientific knowledge; socially responsible practice of the profession, in the best interest of the patients; and professionalism, including ensuring high quality services and ethical practice.

### Austria

In Austria, legal regulations for psychologists were established in 1990 through the Psychologists Act 2013 BGBl. I No. 182/2013, created to legally regulate specialization areas through education, training, and continuing education. The competent authority, the Ministry of Health, is figuratively responsible for the content and makes use of an expert committee, the Psychologists’ Advisory Council, which advises the minister on specialist issues. With the help of experts from science and practice, this psychologist advisory board has, in turn, worked out the content of the training to become a clinical psychologist (the basis for the specialty of clinical neuropsychology), as well as further training in areas of specialization, such as clinical neuropsychology. These sets of criteria (Psychologist Advisory Board of the Minister of Health, 2016) are binding for all providers of specializations, as ultimately, the Ministry of Health also registers the specialization in the official list of the Federal Ministry of Health and Women (2013)^5^. The Criteria Catalogue for Clinical Neuropsychology contains the following fields of activity as areas in which competency is required: knowledge and skills pertaining to neuropsychological profiles of disorders, handling diagnostic questions, implementing diagnostic procedures and treatment approaches and methods, and framework conditions for full-time professional activity over several years of supervision and further training. This full-time training must be completed in multi-professional facilities, such as neurological, neurosurgical, and psychiatric departments in hospitals, specialized rehabilitation facilities, and other similar contexts.

After obtaining the master’s degree, the Austrian model requires the completion of training as a clinical psychologist, which according to §§23 and 24 Psychologengesetz 2013 dictates the practical (2,098 h) and theoretical (340 units) training, plus case supervision (120 units) and self-awareness (76 units). Here, too, the content and core competencies are decisively stated in the law. The additional training for the specialty area follows the structure of becoming a clinical psychologist and requirements are based on §29. This states that “*…after obtaining the professional license, corresponding psychological scientifically based knowledge as well as skills can be proven, which were acquired in particular within the framework of several years of professional focus-specific activity and a further training curriculum comprising at least 120 units. …Designations are permissible which correspond to focal subjects [specializations] within the framework of the study of psychology or which already characterize previous professional focal points.*” The practice period is 2 years of full-time work. The Psychology Advisory Board has the authority to establish new areas of specialty without this necessitating a change in the law.

### Activities of the Standing Committee on Clinical Neuropsychology

In 2015, EFPA formed a task force charged with the mission to clearly define the specialty area of clinical neuropsychology, including the training and competencies necessary to practice. This task force later evolved within EFPA into the Standing Committee on Clinical Neuropsychology (SCCN) (European Federation of Psychologists’ Associations, 2015a), highlighting the firm commitment of EFPA to work toward establishing clinical neuropsychology as a distinct specialty area of psychology.

To date, the SCCN has conducted a series of surveys throughout European countries (Hokkanen et al., 2019, 2020) to ascertain the status of the profession within Europe. Findings from these surveys identified considerable variability among the member states with respect to the training of specialists and the practice of the profession, although representatives from all member states agreed that a European universal certification or license to practice clinical neuropsychology would be desirable.

Given the significant differences in training standards and required qualifications for the speciality area of clinical neuropsychology among European countries, the SCCN has focused its efforts on proposing minimum core competencies for clinical neuropsychologists. Such a proposal would allow flexibility in obtaining the minimum required competencies through various pathways (i.e., via formal education, non-formal training, supervised practice, or a combination of the above) and at various levels of education (e.g., master’s degree, doctoral degree, and postdoctoral level) in each country. Such flexibility is critical in any proposal, as complete uniformity in training would be impossible to achieve, given that formal education in psychology is to some extent already established or even dictated by law in the majority of the member states.

Specifically, the SCCN operated within three important parameters: (1) to form a European definition of Clinical Neuropsychology; (2) to define entry level academic criteria for training in clinical neuropsychology informed by a European survey; and (3) to identify the minimum acceptable competencies required for practicing clinical neuropsychology, based on surveys completed by experienced clinical neuropsychologists in scientific and applied fields within Europe. In accordance with the first of the aforementioned parameters, the definition of clinical neuropsychology proposed by the SCCN (as submitted to EFPA on October 11, 2018^6^) (European Federation of Psychologists’ Associations, 2018) is as follows:

*Clinical Neuropsychology is the application of neuropsychology in clinical situations across the life span. The activity of Clinical Neuropsychology is directed at inferring alterations in the cognitive and affective functioning of the individual and how these relate to the nervous system in general and the brain in particular. The practice of Clinical Neuropsychology is fundamentally based on the exploration and systematic evaluation with the use of psychological and neuropsychological assessment instruments, along with other sources of information. A clinical case formulation thus produced is used both for diagnostic and prognostic purposes, which contribute to: (1) planning, (2) implementation and (3) evaluation of neuropsychological intervention.*

*A Clinical Neuropsychologist draws upon scientific and technical knowledge comprising the skilled use of clinical interview techniques and standardized instruments. The practice of Clinical Neuropsychology demands at a minimum: formal knowledge of psychometry; psychopathology; neurobiological correlates of normal and abnormal cognitive functioning and affective mood. Consideration is given to how such phenomena are expressed in different stages of development and in context of the individual, family, and society. The Clinical Neuropsychologist, in the context of normal functioning and acquired and developmental brain injury, will apply to the individual needs, such skills to understanding and implementing individual and group interventions. This may include behavior management or modification, psychoeducation, and psychological support, as well as the implementation of programs involving stimulation, maintenance, or rehabilitation of neuropsychological functions.*

In a recent investigation of the second parameter, entry-level academic criteria required for the specialty of clinical neuropsychology, the SCCN found that three countries (Austria, Netherlands, and Portugal) regulate the education and/or practice, as well as the title of clinical neuropsychologist (or advanced specialization in neuropsychology in the latter) through legal statutes (Hokkanen et al., 2019). The most common university degree reported to be required for the practice of clinical neuropsychology was the master’s degree; a doctoral degree was required in two countries (the United Kingdom and Ireland; Hokkanen et al., 2019). Where required for the practice of any specialty in psychology, the duration of post master’s level education/training varied from 12 to 60 months. In one third of the countries, no commonly agreed upon model for educating specialists in clinical neuropsychology existed. Countries with more systematic training models and a longer duration of training also tended to report a high level of independence in the work of clinical neuropsychologists (e.g., determining diagnoses, developing treatment plans).

The third of the parameters of the work of the SCCN related to the identification of common core competencies for clinical neuropsychologists in Europe and is the focus of the present study. To delineate these factors, we explored relevant foundational competencies, functional competencies (both knowledge-based and applied) specific to clinical neuropsychology, and additional functional competency areas that are relevant, but not limited, to clinical neuropsychology. We expected to find a high degree of uniformity in perspectives, if not actual training models and practice.

## Materials and Methods

### Participants

Participants were clinical neuropsychologists from 28 European countries with 10 or more years of work experience in clinical and/or scientific practice and thorough knowledge of the educational and training system in their respective countries. The countries represented were Austria, Belgium, Croatia, Cyprus, Czechia, Denmark, Estonia, Finland, France, Germany, Greece, Iceland, Ireland, Italy, Latvia, Lichtenstein, Lithuania, Luxembourg, Norway, Poland, Portugal, Russia, Serbia, Spain, Sweden, Netherlands, Turkey, and the United Kingdom. Two senior clinical neuropsychologists were interviewed from each country and the consensus of their responses was entered into the dataset.

### Procedure

A member of the SCCN of EFPA arranged for the interviews and collected the information into a country-based consensus. Background information involved the names of the interviewer and the respondents. The interview was organized into three parts (form included in the Supplementary Appendix). The first part was based on the framework used in the determination of the specialty area of clinical neuropsychology in the United States^7^ (2012) and was divided into three subsections: (1) foundational competencies (scientific knowledge and methods, individual and cultural diversity, ethical, legal standards and policy, professionalism, reflective practice, relationships, interdisciplinary systems, and evidence-based practice – EBP); (2) functional, knowledge-based and applied competencies unique to clinical neuropsychology (assessment, intervention, and consultation); and (3) additional functional competencies relevant to clinical neuropsychology (research/evaluation, teaching/supervision, management/administration, and advocacy). The interviewees were asked to indicate if and how important they considered a list of competencies for entering the profession (yes, very important; yes, somewhat important, and no, not important). The competencies listed could have been achieved at any point in a person’s education or training.

In the second part of the interview, questions pertained to the methods currently used in each country to determine that the required competencies had been attained (specifically, theoretical coursework, practical training, formal certification or board exam, case logs or description or portfolio, thesis, and a final paper). These were yes/no questions with an opportunity to elaborate if necessary.

Finally, the third part of the interview reflected opinions regarding the importance of a series of statements on education and training in each country, based on a 2017 survey (Hokkanen et al., 2019). Participants were asked to indicate their support (yes, agree fully; agree, in part; no, do not agree) for statements regarding the importance of specialist education being preceded by a minimum of a 5-year master’s level training plus 1 year practicum; the importance of including theoretical courses, supervised practical training, and research; the importance of having any theoretical training (whether within a program or individual coursework) be accredited by a national authority; the importance of sufficient duration, depth, and breadth of the training so as to allow for advanced competence to develop before entering the profession; and whether or not the EFPA specialty in clinical neuropsychology should be equivalent in terms of its criteria to those of other certified specialty areas (namely, psychotherapy and work and organizational psychology). Only descriptive statistics were conducted, given the nature of the data collected and the purpose of the present study.

## Results

Based on our interview, we found a high degree of concurrence overall on views regarding many points related to training and competencies, but to a lesser degree with respect to specific academic requirements. Interview responses indicated that a minimum academic qualification for becoming a clinical neuropsychologist is a master’s degree in 20 (71.4%) countries, a doctorate in two (7.1%) countries, and a bachelor’s degree in one (3.6%) country.

### Foundational Competencies Relevant to Clinical Neuropsychology, but Common Across Functional Domains

Part 1 of the interview explored the interviewees’ ratings of the level of importance of various foundational competencies relevant to clinical neuropsychology that are common across functional domains and to what extent they should be expected from a clinical neuropsychologist entering the profession (after completing specialist education and training). In most countries (76%), knowledge of relevant scientific issues and methods, ethical concepts and legal issues in healthcare, professional identity and awareness of the roles of clinical neuropsychologists, and issues related to relationships and communication with patients, families, and caregivers were acknowledged as important, with 72% endorsing knowledge regarding individual and cultural diversity, the limits of competence and setting goals to improve skill level, communication within interdisciplinary teamwork, and EBP.

More specifically, with respect to EBP, many indicated as important knowledge of assessment strategies and test selection (80%), the scientific basis for diagnostic conclusions in neuropsychological disorders (76%), key symptoms and the way in which relevant disease processes are expressed, decision-making strategies in differential diagnosis, and age-related changes across the lifespan (72%), as well as incidence, prevalence, and the natural course of relevant disorders (68%). Fewer interviewees indicated the importance of knowledge regarding the application of outcome research as a guide for assessment and intervention, and of an EBP approach to assessment and intervention (64%), as well as the application of information technology in the evaluation of best evidence (50%). Table 1 lists percentages of countries in which the aforementioned variables were viewed as somewhat or very important (missing values indicate that there was no response from either of the experts interviewed).

**TABLE 1:** Percentage of respondents giving an affirmative or negative response on items pertaining to foundational competencies relevant, but not limited to, clinical neuropsychology: ratings given as yes, very important, yes, somewhat important or no, not important.

Clusters of competencies	Specific competencies	Yes, very important, % (*n*)	Yes, somewhat important, % (*n*)	No, not important, % (*n*)	Missing, % (*n*)
Scientific knowledge and methods	Clinical and cognitive neurosciences and other relevant fields	85.7 (24)	10.7 (3)	0 (0)	3.6 (1)
Individual and cultural diversity	Diversity integrated in the process of assessment and interpretation of results	50.0 (14)	46.4 (13)	0 (0)	3.6 (1)
Ethical, legal standards and policy	Ethical concepts and legal issues in healthcare, school, military/veteran, industry, forensic etc. including, e.g., informed consent, third party assessments, test security	67.9 (19)	28.6 (8)	0 (0)	3.6 (1)
Professionalism	Professional identity and awareness of the roles of clinical neuropsychologists	75.0 (21)	21.4 (6)	0 (0)	3.6 (1)
Reflective practice	Limits of competence, goal of improving skill level	78.6 (22)	17.9 (5)	0 (0)	3.6 (1)
Relationships	Relationships and communication with patients, families, caregivers etc.	92.9 (26)	3.6 (1)	0 (0)	3.6 (1)
Interdisciplinary systems	Knowledge of and communication within interprofessional teamwork	82.1 (23)	10.7 (3)	3.6 (1)	3.6 (1)
**Evidence-based**					
**practice (EBP)**	Knowledge of key symptoms and expressions of relevant disease processes	89.3 (25)	7.1 (2)	0 (0)	3.6 (1)
	Knowledge of age-related changes across the lifespan	71.4 (20)	25.0 (7)	0 (0)	3.6 (1)
	Knowledge of basis for assessment strategy, test selection	85.7 (24)	10.7 (3)	0 (0)	3.6 (1)
	Knowledge of incidence, prevalence, natural course of relevant conditions	60.0 (16)	37.0 (10)	3.6 (1)	0 (0)
	Knowledge of decision-making, strategies in different diagnoses	75.0 (21)	21.4 (6)	0 (0)	3.6 (1)
	Knowledge of scientific basis for diagnostic conclusions in neuropsychological disorders	92.9 (26)	3.6 (1)	0 (0)	3.6 (1)
	Application of outcome research as a guide for assessment and intervention	60.7 (17)	28.6 (8)	7.1 (2)	3.6 (1)
	Application of EBP components in assessment and intervention	67.9 (19)	25.0 (7)	7.1 (2)	3.6 (1)
	Application of information technology in evaluation of best evidence	25.0 (7)	57.1 (16)	10.7 (3)	7.1 (2)

### Functional Knowledge-Based and Applied Competencies Unique to Clinical Neuropsychology

With respect to functional knowledge-based and applied competencies unique to clinical neuropsychology, ratings were quite consistent. Regarding knowledge-based assessment, most of those who responded indicated that the following areas of knowledge relevant to assessment are important for practicing clinical neuropsychology: 83% indicated theories and methods of measurement and psychometrics; 79% patterns of impairment in neurological disease and the influence of motivational factors and the assessment context; 75% the neuropsychology of behavior and patterns of impairment in psychiatric disorders; 71% neurodiagnostic techniques, medications and their effects on brain functioning and behavior, and the functional implications of impairment; 61% the effects of systemic medical illness on brain functioning and behavior; and 50% on neurochemistry, neurophysiology, and neuroendocrinology. Regarding knowledge-based interventions, 79% endorsed the importance of training in evidence-based intervention practices and the theoretical and procedural bases of interview methods, 75% the effects of neurobehavioral disorders and sociocultural factors on intervention, the identification of intervention targets and needs, and the implementation of interventions, 71% assessment and feedback for therapeutic benefit, development and implementation of a treatment plan, and the evaluation of intervention effectiveness, 67% intervention provided by other professionals and awareness of ethical and legal ramifications of interventions, 62.5% activities for the promotion of cognitive health and identification of barriers to intervention.

When asked about the importance of functional competencies regarding applied aspects of the assessment and intervention processes, we found agreement among most interviewees on most factors related to assessment: 83% indicated that the analysis of the referral question, the gathering of information, the selection, administration and scoring of tests and measures, the interpretation of the results, the formation of an integrative conceptualization, and written communication skills in the production of the assessment report were important; 75% rated recommendations for management and providing feedback adapted to specific audiences as important; and only 58% judged addressing issues related to special populations to be important components of training. Also, there was a high percentage of concurrence on items related to the applied aspect of intervention. Most judged as important the training in the identification of intervention targets and needs, the implementation of interventions (75%), the assessment and feedback for therapeutic benefit, the development and implementation of a treatment plan, the evaluation of intervention effectiveness (71%), the awareness of the ethical and legal ramifications of interventions (67%) and the identification of barriers to intervention (63%).

Questions regarding types of functional knowledge and applied skills regarding consultation also yielded a high percentage of concurrence, but lower than the aforementioned components. Specifically, most said that training in the following areas is important: communication of findings from a consultation, providing assessment feedback and recommendations, and providing consultation services in clinical practice (67%), determination and clarification of referral issues, education of referral sources regarding neuropsychological services, communication of scientific findings (62.5%), knowledge of professional roles and expectations (58%), providing consultation in clinical research (57%), knowledge of relevant literature (54%), and methods of consultation (52%). Table 2 lists the aforementioned variables and the percentages of interviewees who considered them somewhat or very important.

**TABLE 2:** Percentage of respondents giving an affirmative or negative response on items pertaining to foundational knowledge-based and applied competencies unique to clinical neuropsychology: ratings given as yes, very important, yes somewhat important, or no, not important.

Clusters	Competencies	Yes, very important, % (*n*)	Yes, somewhat important, % (*n*)	No, not important, % (*n*)	Missing, % (*n*)
Assessment (knowledge-based)	Neuropsychology of behavior	92.9 (26)	3.6 (1)	0 (0)	3.6 (1)
	Patterns of impairments in neurological diseases	78.6 (22)	17.9 (5)	0 (0)	3.6 (1)
	Neurochemistry, neuropsychopharmacology, neuroendocrinology	50.0 (14)	32.1 (9)	10.7 (3)	7.1 (2)
	Neurodiagnostic techniques	60.7 (17)	28.6 (8)	3.6 (1)	7.1 (2)
	Effects of systemic medical illnesses on brain functioning and behavior	67.9 (19)	25.0 (7)	3.6 (1)	3.6 (1)
	Patterns of impairments in psychiatric disorders	67.9 (19)	28.6 (8)	0 (0)	3.6 (1)
	Influences of motivational factors and assessment context	75.0 (21)	21.4 (6)	0 (0)	3.6 (1)
	Medications and their effects on brain functioning and behavior	46.6 (13)	46.4 (13)	3.6 (1)	3.6 (1)
	Theories and methods of measurement and psychometrics	82.1 (23)	14.3 (4)	0 (0)	3.6 (1)
	Functional implications of impairment	71.4 (20)	21.4 (6)	0 (0)	7.1 (1)
Assessment (applied)	Analysis of the referral question	75.0 (21)	21.4 (6)	0 (0)	3.6 (1)
	Gathering of information	85.7 (24)	10.7 (3)	0 (0)	3.6 (1)
	Selection of tests and measures	92.9 (26)	3.6 (1)	0 (0)	3.6 (1)
	Administration and scoring the tests and measures	89.3 (25)	7.1 (2)	0 (0)	3.6 (1)
	Interpretation of results, formation of an integrated conceptualization	96.4 (27)	0 (0)	0 (0)	3.6 (1)
	Recommendations for management	60.7 (17)	32.1 (9)	0 (0)	7.1 (2)
	Written communication skills in production of assessment report	75.0 (21)	21.4 (6)	0 (0)	3.6 (1)
	Providing of feedback, adapted to specific audiences	71.4 (20	25.0 (7)	0 (0)	3.6 (1)
	Addressing issues related to specific populations	53.6 (15)	39.3 (11)	3.6 (1)	3.6 (1)
Intervention (knowledge-based)	Evidence-based intervention practices	71.4 (20)	25.0 (7)	0 (0)	3.6 (1)
	Theoretical and procedural bases of intervention methods	85.7 (24)	10.7 (3)	0 (0)	3.6 (1)
	Effects of neurobehavioral disorders and sociocultural factors on interventions	71.4 (20)	25.0 (7)	0 (0)	3.6 (1)
	Activities for promoting cognitive health	57.1 (16)	28.6 (8)	10.7 (3)	3.6 (1)
	Interventions provided by other professionals	32.1 (9)	57.1 (16)	7.1 (2)	3.6 (1)
Intervention (applied)	Identification of intervention targets and needs	78.6 (22)	17.9 (5)	0 (0)	3.6 (1)
	Assessment and feedback for therapeutic benefit	71.4 (20)	25.0 (7)	0 (0)	3.6 (1)
	Identification of barriers to intervention	67.9 (19)	28.6 (8)	0 (0)	3.6 (1)
	Development and implementation of a treatment plan	78.6 (22)	14.3 (4)	3.6 (1)	3.6 (1)
	Implementation of interventions	71.4 (20)	25.0 (7)	0 (0)	3.6 (1)
	Evaluation the effectiveness of the intervention	78.6 (22)	17.9 (5)	0 (0)	3.6 (1)
	Awareness of ethical and legal ramifications of intervention	75.0 (21)	21.4 (6)	0 (0)	3.6 (1)
Consultation (knowledge-based)	Professional roles and expectations	60.7 (17)	32.1 (9)	0 (0)	7.1 (2)
	Relevant literature	67.9 (19)	25.0 (7)	0 (0)	7.1 (2)
	Methods of consultation	57.1 (16)	35.7 (10)	0 (0)	7.1 (2)
Consultation (applied)	Determination and clarification of referral issues	57.1 (16)	39.3 (11)	0 (0)	3.6 (1)
	Education of referral sources regarding neuropsychological services	60.7 (17)	35.7 (10)	0 (0)	3.6 (1)
	Communication of findings from consultation	75.0 (21)	21.4 (6)	0 (0)	3.6 (1)
	Providing assessment feedback and recommendations	78.6 (22)	17.9 (5)	0 (0)	3.6 (1)
	Providing consultation services in clinical practice	71.4 (20)	25.0 (7)	0 (0)	3.6 (1)
	Communication of scientific findings	53.6 (15)	42.9 (12)	0 (0)	3.6 (1)
	Providing consultation in clinical research	42.9 (12)	50.0 (14)	0 (0)	7.1 (2)

### Additional Functional Competency Areas Relevant to Clinical Neuropsychology (Research/Evaluation, Teaching/Supervision, Management/Administration, and Advocacy)

Additional questions addressed functional competency areas relevant, but not specific, to clinical neuropsychology. Several items referred to the requirement that specialists have acquired functional knowledge about research and teaching. Specifically, 79% said that knowledge of ethical standards in the conduction of research and knowledge of national and institutional guidelines is important, 75% indicated the importance of training in the scientific method to generate knowledge and evaluate findings, as well as knowledge regarding research design and analysis, and 67% reported knowledge of the array of factors that mediate and modulate behavior and their implications for neuropsychological research to be important. With respect to applied skills in the realm of research/evaluation, 71% endorsed the importance of training in approaches to selecting research topics and performing literature reviews, and 62% endorsed the importance of training in communicating research findings, demonstrating skills in conceptualization, implementation, and interpretation of research designs and conducting statistical analyses, as well as performing research activities, monitoring progress, and evaluating outcomes.

When asked about knowledge-based competency and applied skills relative to teaching/supervision, a little over half the sample rated these areas as important. More specifically, in terms of knowledge-based competency, 54% rated supervision theories, methods, and practices in professional psychology and clinical neuropsychology, as well as ethical issues and national requirements relevant to teaching and supervision as important, and 50% developmental strategies in training. With respect to applied skills which were judged to be important, effective training of psychology trainees was endorsed relative to the foundations of assessment (63%), the neuropsychological interview, test interpretation, case conceptualization, and development of recommendations (58%), the development and assertion of a professional identity and role (57%), treatment planning and provision of feedback (54%), effective teaching, presentation of materials in an organized manner appropriate to the audience (50%), and sensitivity to individual and cultural differences in the supervisory context (46%).

Functional knowledge related to management, administration and advocacy was generally not deemed to be an important part of training by the majority of interviewees. Of the respondents, 42% indicated knowledge of methods and procedures for outcome assessment, program evaluation and research as important, with only 38% rating knowledge of administrative structures in practice settings, 35% knowledge regarding regulatory and policy initiatives that can affect the provision of neuropsychological services and access to care, and 29% common administrative business practices, as important. Applied skills related to management, administration and advocacy were also not endorsed by the majority of the respondents as core competencies: skills related to collaborating with psychologists and other professionals to advocate for neuropsychology (63%); skills related to functioning effectively within administrative systems (42%); educating the public (39%); and implementing administrative structures to address needs in neuropsychological settings, as well as training and supervising technicians/psychometrists or other related professionals and monitoring their skills (25%).

An open-ended question was posed regarding other potential competencies that the interviewees considered relevant for a neuropsychologist entering the profession that had not been included in the list. This yielded the following responses: activities for the promotion of emotional health; a foreign language (specifically, Russian in Estonia); knowledge of national laws and procedures; knowledge of service development and understanding the role of family dynamics in clinical formulations; capacity assessments and planning, as well as studies related to professional experience in clinical investigations; psychological evaluations and neuropsychological testing. Table 3 lists the aforementioned variables and the percentages of interviewees considering them to be somewhat or very important.

**TABLE 3:** Percentage of respondents giving an affirmative response on items pertaining to additional functional competency areas relevant to clinical neuropsychology (research/evaluation, teaching/supervision, management/administration, and advocacy): ratings given as somewhat important, very important or not important.

Clusters	Competencies	Yes, very important, % (*n*)	Yes, somewhat important, % (*n*)	No, not important, % (*n*)	Missing, % (*n*)
Research/evaluation (knowledge-based)	The scientific method in generating knowledge and evaluating findings	67.9 (19)	25.0 (7)	0 (0)	7.1 (2)
	Research design and analysis	60.7 (17)	32.1 (9)	0 (0)	7.1 (2)
	The array of factors that mediate and modulate behavior and their implications for neuropsychological research	60.7 (17)	32.1 (9)	0 (0)	7.1 (2)
	Ethical and responsible manner to perform research, national and institutional guidelines	64.3 (18)	28.6 (8)	0 (0)	7.1 (2)
Research/evaluation (applied)	Select research topics and perform literature reviews	50.0 (14)	39.3 (11)	0 (0)	10.7 (3)
	Demonstrate skills in conceptualizing, implementing, and interpreting research design and statistical analysis.	50.0. (14)	39.3 (11)	3.6 (1)	7.1 (2)
	Perform research activities, monitoring of progress, and evaluation of outcomes	57.1 (16)	28.6 (8)	3.6 (1)	10.7 (3)
	Communicate research findings	53.6 (15)	28.6 (8)	7.1 (2)	10.7 (3)
	Apply research methods in evaluating effectiveness of professional activities	53.6 (15)	25.0 (7)	7.1 (2)	14.3 (4)
Teaching/supervision (knowledge based)	Supervision theories, methods, and practices in professional psychology and clinical neuropsychology	60.7 (17)	25.0 (7)	7.1 (2)	7.1 (2)
	Developmental stages in training	39.3 (11)	39.3 (11)	10.7 (3)	10.7 (3)
	Ethical issues and national requirements relevant to teaching and supervision	64.3 (18)	21.4 (6)	7.1 (2)	7.1 (2)
Teaching/supervision (applied)	Effective teaching, presenting materials in an organized manner appropriate to the audience	42.9 (12)	39.3 (11)	10.7 (3)	7.1 (2)
	Effective training to psychology trainees in the foundations of assessment	46.4 (13)	35.7 (10)	7.1 (2)	10.7 (3)
	Effective training in developing and asserting professional identity and role	46.4 (13)	35.7 (10)	7.1 (2)	10.7 (3)
	Effective training in neuropsychological interviewing, test interpretation, case conceptualization, and the development of recommendations	71.4 (20)	14.3 (4)	7.1 (2)	7.1 (2)
	Effective training in treatment planning and the provision of feedback	64.3 (18)	17.9 (5)	10.7 (3)	7.1 (2)
	Sensitivity to individual and cultural differences in supervisory contexts	46.4 (13)	35.7 (10)	10.7 (3)	7.1 (2)
Management, administration (knowledge-based)	Administrative structures of practice settings	39.3 (11)	42.9 (12)	14.3 (4)	3.6 (1)
	Common administrative and business practices	25.0 (7)	46.4 (13)	17.9 (5)	10.7 (3)
	Methods and procedures for outcome assessment, program evaluation, and research	39.3 (11)	35.7 (10)	14.3 (4)	10.7 (3)
Management, administration (applied)	Function effectively within administrative systems	28.6 (8)	46.4 (13)	7.1 (2)	17.9 (5)
	Implement administrative structures to address needs in neuropsychology practice settings	32.1 (9)	39.3 (11)	14.3 (4)	14.3 (4)
	Train and supervise technicians/psychometrists or other related professionals and monitor their skills	28.6 (8)	28.6 (8)	25.0 (7)	17.9 (5)
Advocacy (knowledge-based)	Regulatory and policy initiatives that can affect provision of neuropsychology services and access to care	32.1 (9)	46.4 (13)	10.7 (3)	10.7 (3)
Advocacy (applied)	Advocate for needs of individuals/groups across systems	25.0 (7)	57.1 (16)	10.7 (3)	7.1 (2)
	Collaborate with psychologists and other professionals to advocate for neuropsychology	50.0 (14)	42.9 (12)	0 (0)	7.1 (2)
	Educate the public	32.1 (9)	46.4 (13)	7.1 (2)	14.3 (4)

### Evaluation/Approval Procedures Currently in Use in Measuring the Competency Level of Clinical Neuropsychologists, Who Complete Their Training and Enter the Profession

Part 2 of the interview explored current practices with respect to assessing competency in new professionals. When asked about the current requirements in each country, most respondents (75%) stated the satisfactory completion of theoretical courses, with 4% of the sample indicating the requirement of both theoretical courses and exams, but 18% said neither was required (3% gave no response). Similarly, 71% indicated that satisfactory completion of practical training was required, with 29% indicating that it was not. A formal certification or board examination was required in 46% of the countries, not required in 46% (8% gave no response). Of those who replied in the affirmative, 15% indicated that a written exam was required, 8% an oral exam, and 54% a combination of the two (23% did not specify). Most responded ‘yes’ when asked if the presentation of a case description, log, or a portfolio was required (57% ‘yes’ and 39% ‘no’). The requirement of a thesis was indicated by 43% but was not standard in most of the countries (57%). Where it was required, 73% indicated a master’s level thesis and 27% a doctorate in psychology. To the question regarding what this thesis would involve (empirical study, meta-analysis, or systematic or non-systematic literature review), 43% indicated an empirical study in any of these categories, and 43% added that a case study would be acceptable. When asked if a specified number of published or unpublished papers is required, most of the participants of the total sample (86%) did not respond, 7% responded that published papers were not required, while 11% said that unpublished papers were not required, with only 4% responding ‘yes’ to the requirement of unpublished papers. With respect to the requirement of unpublished papers, 86% indicated that two are required, 4% said one is required, 4% said there is no such requirement, and 4% merely indicated that there is a requirement without specifying the number. To a question regarding whether or not there is a requirement of submitting a final paper (including the following formats: empirical, meta-analysis, systematic or non-systematic literature review), 50% said ‘yes’ and 46% said ‘no’ (4% gave no response).

In the final part of the interview (Part 3), the following question was posed: “In reference to the specialist education and training in clinical neuropsychology, would you support the following statements?” Respondents mostly agreed that specialist training should be preceded by at least a 5-year master’s degree (or equivalent) in psychology and at least a minimum of 1-year clinical practice: 78% fully agreed, 18% partially agreed, and 4% did not agree. In response to the question of whether the core element of specialist education should include theoretical study, practical training with supervision, and research experience, 93% agreed fully, with 7% agreeing partially. When asked if they thought that theoretical studies, whether in the form of a program or a combination of separate courses, should be accredited by a national authority, 89% agreed fully and 11% partially. Importantly, none disagreed on these last questions.

Another question referred to the length, depth, and breadth of the different elements within the specialist education being sufficient to allow for the accumulation of advanced competencies necessary for a successful entry into the profession. Specifically, the question was whether they thought that achieving these competencies typically requires several years of specialization in clinical neuropsychology. To this, 96% agreed fully and 4% partially. Again, none disagreed. Finally, in comparison to existing EuroPsy specialist certification requirements, 73% thought that the specialist certification for clinical neuropsychology should be equivalent to existing criteria for other specialties, 23% indicated their opinion that the criteria should be more demanding than those in existing EuroPsy specialties and 4% said they should be less demanding.

## Discussion

The present study demonstrated a high degree of consensus among experienced clinical neuropsychologists from 28 European countries regarding desirable competencies for those entering the specialty area of clinical neuropsychology, despite many differences in the educational and training systems. Specifically, most respondents confirmed the importance of relevant clinical knowledge and skills, as well as research, with fewer respondents endorsing the importance of administrative/management skills. There was also some degree of similarity among the training programs among the countries.

More specifically, we found that approximately three quarters of the respondents agreed on an array of competency areas, including a strong knowledge base and skills in psychometric theory and methods, neurological and mental disorders, scientific methods, ethics, legal issues and healthcare, professional issues, communication skills with clients and their families, cultural diversity, skills related to working in an interdisciplinary environment, and EBP. More than 70% also endorsed the importance of competence in interviewing and history taking, providing feedback and helpful recommendations following an assessment, and treatment planning and application as integral training and practice competencies for clinical neuropsychologists.

Additionally, most of the experts interviewed in the current study placed value primarily on scientific knowledge (e.g., neuroanatomy, psychometrics, impairments in neurological, and mental disorders) and practical training (e.g., assessment, history taking, intervention, communication of findings) – skills and knowledge that are at the core of a clinical neuropsychologist’s work – as required areas indicative of competency for entry into the profession. Secondly, they valued knowledge and competence in research, ethics, individual and cultural diversity, supervision, and EBP. Moreover, many stated that training programs in their country require a thesis or a final (published or unpublished) paper, thus, highlighting the value imbued upon not only attaining, but also creating, new scientific knowledge. Finally, training in practical work-related aspects, such as knowledge regarding management, administration, and advocacy, were less often endorsed than the aforementioned competencies. Thus, it appears that the experts advocate a traditional scientist-practitioner model. This is in line with the Boulder Model of training in clinical psychology (proposed in 1949; for a history and critique, see Frank, 1984), which is the standard in the United States. Our findings also indicated that management, policy, and advocacy are not central to our perspective of a competent specialist in clinical neuropsychology and may be assumed to be learned on the job and within the relevant professional context.

In most of the countries (specifically, 20), at least a master’s degree is required for the specialization in clinical neuropsychology, a doctorate in two, and a bachelor’s degree in one. The remaining countries either have no training program in clinical neuropsychology or no educational requirements for its practice. Almost all respondents agreed that the training of a clinical neuropsychologist should require several years in order to acquire sufficient theoretical and practical competencies for independent professional practice. A strong majority also agreed that specialist training should begin only after the master’s degree. Finally, about three quarters of the respondents were in favor of developing a European specialist certification for clinical neuropsychology with similar criteria (in terms of number of years of training, hours of supervised practica, etc.) as the existing EuroPsy specialist certifications in Psychotherapy and Work and Organizational Psychology.

The current findings, based on a European sample, are consonant with previous studies exploring the most valued competencies for clinical neuropsychology across continents. Hessen et al. (2018) found common requirements for sound training in foundational, (e.g., scientific knowledge, diversity issues, ethics, and EBP), functional (e.g., psychometric theory, neurodiagnostic, history taking, EBP, and client consultation) and other competencies (e.g., teaching and supervision) to ensure sufficient entry-level expertise in clinical neuropsychology in the United States, Australia, Finland, Italy, Netherlands, Norway, and the United Kingdom. While the present study confirmed previous investigations with respect to the importance of functional and foundational competencies in academic and clinical training, we found a definite gradation with respect to degree of consensus, with few experts endorsing the importance of training in teaching, supervision, and research, and even fewer endorsing training in management, administration, and advocacy as core competencies for entry-level clinical neuropsychologists. The overall pattern of responses did not differ based on whether respondents were from countries with clearly defined and rigorous training models vs. those where such structured training is not required and/or available. Thus, there appears to be a clear consensus in terms of the core nature of the knowledge and skills that would constitute competency, with others judged as secondary.

The strength and informative value of this study is undoubtedly the fact that for the first time it was possible to collect data relating to the content of training and that every European country that has a training association or a professional community of interest was surveyed or included. Regardless of the different histories or underlying cultures or health systems, the core competencies of senior experts were not only assessed, but also compared with existing systems, for example those of North America and Canada. The overlap in the most consistently valued areas of competency reinforces their importance for training of specialists in clinical neuropsychology. Perhaps more importantly, however, is that it may aid countries without specific regulations to build their speciality requirements based on the core competencies reported herein. Additionally, commonalities across Europe may facilitate the mobility of professionals and trainees, as well as both clinical and research collaborations.

Limitations of the present study should be mentioned. Not all EFPA member associations were represented in the survey, as some representatives of national associations did not respond or did not name their experts. Moreover, our findings are based on a panel of experts, specifically two from each member country. Although this is not an uncommon methodology, it may restrict the generalizability of our findings. Additionally, varied experiences in terms of training and professional standards, including longstanding standards in some countries, might have influenced the experts’ perspective on core competencies. Finally, experts were not all a homogeneous group, as they had various professional and/or academic roles for which we did not control.

Clinical neuropsychology is a rapidly growing, internationally recognized specialty area in psychology requiring advanced-level training in distinct areas of knowledge and skills. In fact, it has been the largest division of the APA since 2017^8^ (American Psychological Association, 2017). Moreover, neurocognitive dysfunction related to a variety of medical conditions beyond the traditional neurological and mental disorders which have typically been the focus of neuropsychological services (e.g., post-operative cognitive dysfunction, COVID-19 related cognitive sequelae, attention deficit disorder, and learning disorders) is increasingly becoming apparent, requiring competent professionals for assessment and rehabilitation in a variety of multidisciplinary settings. Furthermore, research in this area is continually refining and expanding our knowledge regarding brain-behavior relationships. As both the need for, and interest in, clinical neuropsychological services increase, and as the scientific field evolves, there may be an influx of individuals with limited competency and/or inadequate training in clinical neuropsychology to function professionally in this field, possibly providing services that are not up to par. For the protection of the public, who are the recipients of these services, as well as the professional field of clinical neuropsychology itself, it behooves us to establish a set of core competencies to ensure that all those who practice in this area have the necessary skills and knowledge to do so effectively. Thus, in addition to a professional issue it is also an ethical issue. Securing minimum universally accepted competences for clinical neuropsychologists across Europe is certainly a step forward in that direction. This could be solidified through the establishment of the advanced EuroPsy certification for clinical neuropsychology as a specialty area based on targeting the skills indicated in the present study as core components of training models for future specialists in clinical neuropsychology.

## Data Availability Statement

The raw data supporting the conclusions of this article will be made available by the authors, without undue reservation.

## Ethics Statement

Ethical review and approval was not required for the study on human participants in accordance with the local legislation and institutional requirements. The patients/participants provided their written informed consent to participate in this study.

## Author Contributions

SL, LH, FB, BP, GB, EK, AmP, SM, NV, TN, EH, and MC: substantial contributions to conception and design of the work. MK, SL, LH, FB, BP, GB, EK, AmP, SM, NV, TN, EH, MC, MJ, and AiP: acquisition of data. MK, SL, LH, FB, BP, GB, EK, AmP, SM, NV, TN, EH, and MC: analysis and interpretation of data. MK, SL, MC, LH, FB, BP, GB, EK, AmP, SM, EH, MJ, and AiP: drafting of manuscript and critically revising. All authors contributed to the article and approved the submitted version.

## Conflict of Interest

The authors declare that the research was conducted in the absence of any commercial or financial relationships that could be construed as a potential conflict of interest.

## Publisher’s Note

All claims expressed in this article are solely those of the authors and do not necessarily represent those of their affiliated organizations, or those of the publisher, the editors and the reviewers. Any product that may be evaluated in this article, or claim that may be made by its manufacturer, is not guaranteed or endorsed by the publisher.

## Footnotes

1
https://www.psykologforeningen.no/medlem/kurs-og-utdanning

2
www.bps.org.uk

3
https://www.royalcollege.ca/rcsite/canmeds/canmeds-framework-e

4
https://www.fgzpt.nl/de-fgzpt/college/thema%E2%80%99s-en-projecten

5
http://klinischepsychologie.ehealth.gv.at/

6
http://clinneuropsy.efpa.eu

7
http://cospp.org/specialties/clinicalneuropsychology

8
https://www.apa.org/about/division/officers/services/profiles
