# Supplementary material for: Core Competencies in Clinical Neuropsychology as a Training Model in Europe
Source: Front Psychol. 2022 Mar 31;13:849151. doi: 10.3389/fpsyg.2022.849151 (PMC9008746; doi:10.3389/fpsyg.2022.849151)
Supplement: Supplementary file 1 [file Data_Sheet_1.docx]

APPENDIX

**Interview form on core competencies in clinical neuropsychology training in European countries**

The interview has three parts. The **first part** is based on the U.S. framework submitted to the Council of Specialties in Professional Psychology (<http://cospp.org/specialties/clinicalneuropsychology>, 2012). This framework was also employed in a recent publication entitled “Core competencies in clinical neuropsychology training across the world” (Hessen et al. 2017). The framework is divided into sections on foundational, functional, and additional competencies. The responders are requested to evaluate how important each of the competencies are when entering the profession of a clinical neuropsychologist in their own countries using a scale 0 = no, does not need to be expected, 1 = yes, is somewhat necessary when entering the profession, 2 = yes, is very necessary when entering the profession. More details on each of the competency items can be found in the original US document. It is important to note that the competency in question may be acquired at any time during the education of a candidate (undergraduate, post-graduate or during supervised clinical work/training). Competencies not mentioned can be added to the list.

The **second part** of the interview is related to the different potential ways to assess the acquired competencies. The **third part** is related to general recommendations regarding education and training of clinical neuropsychologists.

To qualify as a responder, one must be a senior clinical neuropsychologist (preferably at least 10 years of independent clinical practice) with a longstanding and detailed knowledge about the training procedures of clinical neuropsychologists in his or her country. The response for each country shall represent the consensus opinion of the two responders from that country. If no consensus can be reached the responders may seek the opinion of one other senior clinical neuropsychologist with similar qualifications from that country, to reach a conclusion.

| **Country:** | | |  |  |  |
| --- | --- | --- | --- | --- | --- |
| **Responder 1:** | | | | | |
| **Responder 2:** | | | | | |
| **Minimum academic qualification for becoming a clinical neuropsychologist:** | Bachelor’s degree | Master’s degree | | Doctorate | Other: |

**PART ONE**

**How would you rate the importance of the following competencies? Should they be expected of a clinical neuropsychologist when he/she is entering the profession (after completion of specialist education and training)?****0 = no, does not need to be expected
1 = yes, is somewhat necessary when entering the profession**

**2 = yes, is very necessary when entering the profession**

**Foundational competencies relevant to clinical neuropsychology but common across functional domains.**

| **Cluster of competencies** | **Specific competencies** | **Responses**  **0 / 1 / 2** |
| --- | --- | --- |
| **Scientific knowledge and methods** | Clinical and cognitive neurosciences and other relevant fields |  |
| **Individual and cultural diversity** | Diversity integrated in the process of assessment and interpretation of results |  |
| **Ethical, legal standards and policy** | Ethical concepts and legal issues in healthcare,  school, military/veteran, industry, forensic etc. including e.g. informed consent, third party assessments, test security |  |
| **Professionalism** | Professional identity and awareness of the roles of clinical neuropsychologists |  |
| **Reflective practice** | Limits of competence, goal of improving skill level |  |
| **Relationships** | Relationships and communication with patients, families, caregivers etc. |  |
| **Interdisciplinary systems** | Knowledge of and communication within interprofessional teamwork |  |
| **Evidence-Based Practice (EBP)** | Knowledge of key symptoms and expressions of relevant disease processes |  |
|  | Knowledge of age-related changes across lifespan |  |
|  | Knowledge of basis for assessment strategy, test selection |  |
|  | Knowledge of incidence, prevalence, natural course of relevant conditions |  |
|  | Knowledge of decision-making strategies in differential diagnosis |  |
|  | Knowledge of scientific basis for diagnostic conclusions in neuropsychological disorders |  |
|  | Application of outcome research as a guide for assessment & intervention |  |
|  | Application of EBP components in assessment and intervention |  |
|  | Application of information technology (IT) in evaluation of best evidence |  |

**Functional knowledge-based and applied competencies unique to clinical neuropsychology.**

| **Clusters** | **Competencies** | **Responses**  **0 / 1 / 2** |
| --- | --- | --- |
| **Assessment**  **(knowledge-based)** | Neuropsychology of behavior |  |
|  | Patterns of impairments in neurological diseases |  |
|  | Neurochemistry, neuropsychopharmacology, neuroendocrinology |  |
|  | Neurodiagnostic techniques |  |
|  | Effects of systemic medical illnesses on brain functioning and behavior |  |
|  | Patterns of impairments in psychiatric disorders |  |
|  | Influences of motivational factors and assessment context |  |
|  | Medications and their effects on brain functioning and behavior |  |
|  | Theories and methods of measurement and psychometrics |  |
|  | Functional implications of impairment |  |
| **Assessment (applied)** | Analysis of the referral question |  |
|  | Gathering of information |  |
|  | Selection of tests and measures |  |
|  | Administration and scoring the tests and measures |  |
|  | Interpretation of results, formation of an integrated conceptualization |  |
|  | Recommendations for management |  |
|  | Written communication skills in production of assessment report |  |
|  | Providing of feedback, adapted to specific audiences |  |
|  | Addressing issues related to specific populations |  |
| **Intervention  (knowledge based)** | Evidence-based intervention practices |  |
|  | Theoretical and procedural bases of intervention methods |  |
|  | Effects of neurobehavioral disorders and sociocultural factors on interventions |  |
|  | Activities for promoting cognitive health |  |
|  | Interventions provided by other professionals |  |
| **Intervention (applied)** | Identification of intervention targets and needs |  |
|  | Assessment and feedback for therapeutic benefit |  |
|  | Identification of barriers to intervention |  |
|  | Development and implementation of a treatment plan |  |
|  | Implementation of interventions |  |
|  | Evaluation the effectiveness of the intervention |  |
|  | Awareness of ethical and legal ramifications of intervention |  |
| **Consultation  (knowledge based)** | Professional roles and expectations |  |
|  | Relevant literature |  |
|  | Methods of consultation |  |
| **Consultation (applied)** | Determination and clarification of referral issues |  |
|  | Education of referral sources regarding neuropsychological services |  |
|  | Communication of findings from consultation |  |
|  | Providing assessment feedback and recommendations |  |
|  | Providing consultation services in clinical practice |  |
|  | Communication of scientific findings |  |
|  | Providing consultation in clinical research |  |

**Additional functional competency areas relevant to clinical neuropsychology (research/evaluation, teaching/supervision, management/administration, and advocacy).**

| **Clusters** | **Competencies** | **Responses**  **0 / 1 / 2** |
| --- | --- | --- |
| **Research / evaluation**  **(knowledge based)** | The scientific method in generating knowledge and evaluating findings |  |
|  | Research design and analysis |  |
|  | The array of factors that mediate and modulate behavior and their implications for neuropsychological research |  |
|  | Ethical and responsible manner to perform research, national and institutional guidelines |  |
| **Research / evaluation**  **(applied)** | Select research topics and perform literature reviews |  |
|  | Demonstrate skills in conceptualizing, implementing, and interpreting research design and statistical analysis. |  |
|  | Perform research activities, monitoring of progress, and evaluation of outcomes |  |
|  | Communicate research findings |  |
|  | Apply research methods in evaluating effectiveness of professional activities |  |
| **Teaching / supervision**  **(knowledge based)** | Supervision theories, methods, and practices in professional psychology and clinical neuropsychology |  |
|  | Developmental stages in training |  |
|  | Ethical issues and national requirements relevant to teaching and supervision |  |
| **Teaching / supervision**  **(applied)** | Effective teaching, presenting materials in an  organized manner appropriate to the audience |  |
|  | Effective training to psychology trainees in the foundations of assessment |  |
|  | Effective training in developing and asserting professional identity and role |  |
|  | Effective training in neuropsychological interviewing, test  interpretation, case conceptualization, and the development of recommendations |  |
|  | Effective training in treatment planning and the provision of feedback |  |
|  | Sensitivity to individual and cultural differences in  supervisory contexts |  |
| **Management, administration**  **(knowledge based)** | Administrative structures of practice settings |  |
|  | Common administrative and business practices |  |
|  | Methods and procedures for outcome assessment, program evaluation, and research |  |
| **Management, administration**  **(applied)** | Function effectively within administrative systems |  |
|  | Implement administrative structures to address needs in  neuropsychology practice settings |  |
|  | Train and supervise technicians/psychometrists or other related professionals and monitor their skills |  |
| **Advocacy**  **(knowledge based)** | Regulatory and policy initiatives that can affect provision of neuropsychology services and access to care |  |
| **Advocacy**  **(applied)** | Advocate for needs of individuals/groups across systems |  |
|  | Collaborate with psychologists and other professionals to advocate for neuropsychology |  |
|  | Educate the public |  |

**Are there other competencies relevant for a neuropsychologist at entry to the profession, not mentioned here?**

|  |  |
| --- | --- |
|  |  |
|  |  |

**PART TWO**

**Evaluation / Approval procedures currently in use in measuring the competency level of clinical neuropsychologists who complete their training and enter the profession (feel free to elaborate beyond yes/no, and also discuss ideas not in use yet):**

|  | **Currently in use** | | |
| --- | --- | --- | --- |
| **Satisfactory completion of theoretical courses?**  Do they include exams? | **Yes/No:** | | |
| **Satisfactory completion of practical training?**  Does it include supervisors’ ratings? |  | | |
| **Formal certification (board) examination?** | **Yes/No:** | | |
| If yes, please specify format | Written | Oral | Combined oral/written |
| **Case log, case description, portfolios?** | **Yes/No:** | | |
| **Thesis?** | **Yes/No** | | |
| If yes, please specify level: | Master | D.Clin.Psy | PhD |
| What does the thesis involve: Empirical study / meta-analysis / systematic literature review / non-systematic (narrative) review?  Number of required published papers?  Number of required unpublished papers? |  | | |
| **Final paper?**  What does it include: Empirical study / meta-analysis / systematic literature review / non-systematic (narrative) review? | **Yes/No** | | |

**Are there ways of measuring competencies that are currently used in your country, not mentioned above? Are there ideas for development of new assessment methods? Please elaborate as necessary.**

**PART THREE**

**In reference to the specialist education and training in clinical neuropsychology, would you support the following statements?**

**1. The specialist education in clinical neuropsychology should be preceded by at least a five-year master’s degree (or equivalent) in psychology and a minimum of one-year clinical practice.**

Yes, agreed fully
Agreed in part (elaborate below)
No, not agreed

**2. The core elements of the specialist education should include theoretical study, practical training with supervision, and research experience.**

Yes, agreed fully
Agreed in part (elaborate below)
No, not agreed

**3. The theoretical studies, whether in the form of a program or a combination of separate courses, should be accredited by a national authority.**

Yes, agreed fully
Agreed in part (elaborate below)
No, not agreed

**4. The length, depth, and breadth of the different elements within the specialist education must be sufficient to allow for the accumulation of advanced competencies necessary for a successful entry in the profession. Achieving these competencies typically requires several years of specialization in clinical neuropsychology.**

Yes, agreed fully
Agreed in part (elaborate below)
No, not agreed

**5. EFPA Specialist certificate in EuroPsy currently means as follows:**

Psychotherapy: 90 ECTS of further study, of which 400 hours should be devoted to theory, 3 years postgraduate practice, of which 500 hours supervised practice, as well as at least 150 hours of supervision, on average 50 hours per year.

Work & Organisational Psychology: 90 ECTS of further study (2400 hours) which 60 (1600) hours) should be devoted to courses, al least 3 years of postgraduate practice, of which 400 hours per year (in total 1200 hours) is supervised practice, at least 150 hours of supervision (on average 50 per year)

**Would you say the requirements for clinical neuropsychologists should be …**

Lower than these
Similar to these
Higher than these

Elaborate as necessary
